# Correction to: *Austropuccinia psidii*, causing myrtle rust, has a gigabase-sized genome shaped by transposable elements

**DOI:** 10.1093/g3journal/jkac251

**Published:** 2022-09-26

This is a correction to: Peri A Tobias, Benjamin Schwessinger, Cecilia H Deng, Chen Wu, Chongmei Dong, Jana Sperschneider, Ashley Jones, Zhenyan Lou, Peng Zhang, Karanjeet Sandhu, Grant R Smith, Josquin Tibbits, David Chagné, Robert F Park, *Austropuccinia psidii*, causing myrtle rust, has a gigabase-sized genome shaped by transposable elements, *G3 Genes|Genomes|Genetics*, Volume 11, Issue 3, March 2021, https://doi.org/10.1093/g3journal/jkaa015

In the originally published version of this manuscript, the following co-author’s name should read: “Zhenyan Luo” instead of “Zhenyan Lou”. This error has now been corrected online.

